# Lower urinary tract symptoms–Benign prostatic hyperplasia may increase the risk of subsequent inguinal hernia in a Taiwanese population: A nationwide population-Based cohort study

**DOI:** 10.1371/journal.pone.0234329

**Published:** 2020-06-08

**Authors:** Yi-Hsuan Wu, Yung-Shun Juan, Jung-Tsung Shen, Hsun-Shuan Wang, Jhen-Hao Jhan, Yung-Chin Lee, Jiun-Hung Geng

**Affiliations:** 1 Department of Urology, Kaohsiung Municipal Hsiao-Kang Hospital, Kaohsiung, Taiwan; 2 Department of Urology, Kaohsiung Medical University Hospital, Kaohsiung, Taiwan; 3 Kaohsiung Medical University, Kaohsiung, Taiwan; 4 Department of Urology, Kaohsiung Municipal Ta-Tung Hospital, Kaohsiung, Taiwan; University Medical Center Utrecht, NETHERLANDS

## Abstract

**Introduction & objectives:**

It has been suggested that lower urinary tract symptoms—benign prostatic hyperplasia (LUTS-BPH) may be a risk factor for inguinal hernia (IH). The aim of this study was to examine the emergence of a subsequent IH diagnosis in men with and without LUTS-BPH.

**Methods:**

From a database derived from the National Health Insurance Program covering 99% of the population in Taiwan, 22,310 men with LUTS-BPH and 22,310 matched men without LUTS-BPH were identified and followed for IH from 1997 to 2013. Both IH and LUTS-BPH were defined by the ninth revision of the International Classification of Diseases code (ICD9). Subjects younger than 20 years of age and with IH diagnosed before the index date were excluded. We used Cox proportional hazards regression models to estimate hazard ratios (HRs) for subsequent IH, controlling for potential confounders.

**Results:**

Men with and without LUTS-BPH had similar age and comorbidity distributions. During the 10 years of follow-up, 1,303 (5.84%) men with LUTS-BPH and 735 (2.53%) men without LUTS-BPH developed IH. The mean time to IH was 4.02 years and 4.44 years, respectively. After adjusting for age and comorbidities, LUTS-BPH was associated with a two-fold increased risk of IH (HR:2.25, 95% CI = 2.04–2.49).

**Conclusion:**

This nation-wide population-based cohort study showed that LUTS-BPH increased the risk of subsequent IH in a Taiwanese Population.

## Introduction

An inguinal hernia (IH) is a protrusion of abdominal-cavity contents, such as intestines, through the inguinal canal [1]. Some patients are not bothered by annoying symptoms, such as a protruding non-painful mass, so a clinician might choose conservative treatments [2–4]. However, more serious symptoms may appear in about 66% of affected people [1], and these might include discomfort or pain especially with lifting, exercise, coughing or bowel movements [5]. The most terrible event is that intestines are trapped and pinched in the groin or scrotum and cannot be moved back into the abdomen, leading to an incarcerated or even strangulated inguinal hernia. This usually produces severe pain and tenderness of the area and the strangulation of intestines, where the blood supply to part of the intestine is blocked, possibly resulting in bowel perforation [6] or gangrene [7]. It is life-threatening and needs emergent surgery [8].

Risk factors for the development of an IH include inheritance [9], gender [10], age [11], collagen metabolism [12], chronic cough [13], chronic constipation [13], and prostatectomy history, especially in retropubic open prostatectomy [1]. Conversely, obesity decreases the occurrence of inguinal hernia [14]. There are several mechanisms for IH, such as musculo-fascial weakness [15], anatomical variations [16], connective tissue alterations [17, 18] and high intra-abdominal pressure [19]. A study with a large population (1.5 million subjects) indicated that increased cumulative intra-abdominal pressure, like lifting, standing and walking, is related to the formation of IH [20]; furthermore, reducing daily cumulative intra-abdominal pressure could prevent IH surgery by 30% [21].

Benign prostatic hyperplasia (BPH) is one of the most common causes of male lower urinary tract symptoms (LUTS) and occurs with aging [22]. As LUTS-BPH progresses despite medical treatment, post void residual (PVR) and urinary tract infection can be identified requiring appropriate surgical intervention [23]. For a urologist, it is not uncommon to discover the coexistence of inguinal hernia and symptomatic BPH. In 1982, while performing transurethral resection of the prostate, Thompson et al. disclosed inguinal hernia in 20% of men with LUTS-BPH [24]. However, we were unable to confirm the association between LUTS-BPH and IH through the previous reports [25, 26]. The aim of this study was to examine the association between LUTS-BPH and IH in a nationwide population-based cohort study.

## Material and methods

### Data source

The Taiwan National Health Insurance (NHI) scheme covers 99% of the population in Taiwan [27]. A database, the Longitudinal Health Insurance Database 2005 (LHID 2005), was released by Taiwan NHI for research purposes [28]. The LHID2005 includes about 1 million beneficiaries drawn randomly from a total of 23 million individuals in the NHI registry of 2005. This database contains all the medical claims of individuals enrolled from 1997 to 2013, and was proven to be statistically identical to the whole population of 23 million individuals in gender distribution (http://nhird.nhri.org.tw/en/Data_Subsets.html). The diagnoses in the LHID 2005 are coded on the basis of the International Classification of Diseases, Ninth Revision, Clinical Modification (ICD-9-CM). The data from LHID2005 is de-identified and encrypted for patient protection. Ethical approval for the study was provided by the Institutional Review Board of Kaohsiung Medical University Hospital (number KMUHIRB-E(I)-20190325).

### Study cohort and information collection

This is a population-based cohort study, including men with LUTS-BPH and a matched comparison cohort of men without LUTS-BPH. All medical claims of men aged 20 and above from 1997–2013 year were collected. We used the ninth revision of the International Classification of Diseases (ICD9) codes 600.XX to identify LUTS-BPH. A diagnosis of LUTS-BPH was based on the clinical symptoms and/or digital examinations by physicians. The date of the first medical visit due to LUTS-BPH was defined as the index date. IH was identified by ICD9 diagnostic codes (ICD9 550.1, 550.1 and 550.9). Men in the comparison cohort were randomly selected from the LHID 2005 and matched to men with LUTS-BPH by age at a 1:1 ratio. Age and past history of hypertension (ICD9 401–405), kidney disease (ICD9 585, 588), diabetes (ICD9 250), liver disease (ICD 570, 571), obesity (ICD9 278), chronic obstructive pulmonary disease (ICD9 491–493, 496) and urethral disease (ICD9 598) were collected for men with and without LUTS-BPH. Subjects younger than 20 years of age, who had received radical prostatectomy (NHI surgical orders codes: 79403B and 79410B) and had been diagnosed with IH before the index date were excluded.

### Statistical analysis

All men were followed from the date of diagnosis until death, emigration or end of follow-up (31 December 2013), whichever event came first. Statistical analyses were performed using SPSS software, version 20.0 (SPSS Inc., Chicago, IL). Percentages were calculated for categorical variables. Chi-square test or Fisher’s exact test were used to analyze the differences between categorical variables. Baseline characteristics such as age and selected comorbidities were considered risk factors for the diagnosis of IH. We included hypertension [29], kidney disease [30], diabetes mellitus [31], liver disease [32], obesity [33], chronic obstructive pulmonary disease [34] and urethral disease [35] as potential confounders. Log-rank tests were used to compare differences in the cumulative incidence of IH between men with and without LUTS-BPH. Cox proportional hazards regression models were applied to estimate hazard ratios (HRs) and 95% confidence interval (CI) for subsequent IH, while controlling for the above-mentioned potential confounders. A two-tailed p value of 0.05 or less was considered statistically significant.

## Results

This study included 22,310 men diagnosed with LUTS-BPH and 22,310 matched comparison men without LUTS-BPH. Table 1 shows the age and comorbidity distributions of all men. Most individuals in our study aged 40 years or above. No significant differences in age and comorbidities were found between men with and without LUTS-BPH.

**Table 1:** Demographic data (N = 44,620).

	LUTS-BPH(-) (n = 22,310)		LUTS-BPH(+) (n = 22,310)		
	N	(%)	N	(%)	p value
Age					
<40	696	(3.1)	699	(3.1)	0.359
40–59	10627	(47.6)	10513	(47.1)	
> = 60	10987	(49.2)	11098	(49.7)	
Comorbidities					
Diabetes	4255	(19.1)	4264	(19.1)	0.914
Hypertension	9840	(44.1)	9819	(44.0)	0.841
Chronic kidney disease	2123	(9.5)	2125	(9.5)	0.974
Liver disease	4532	(20.3)	4454	(20.0)	0.357
Obesity	133	(0.6)	123	(0.6)	0.531
COPD	7642	(34.3)	7641	(34.2)	0.992
Urethral disease	51	(0.2)	51	(0.2)	>0.999

LUTS-BPH, lower urinary tract symptoms—benign prostate hyperplasia; COPD, chronic obstructive pulmonary disease.

**Fig 1:**
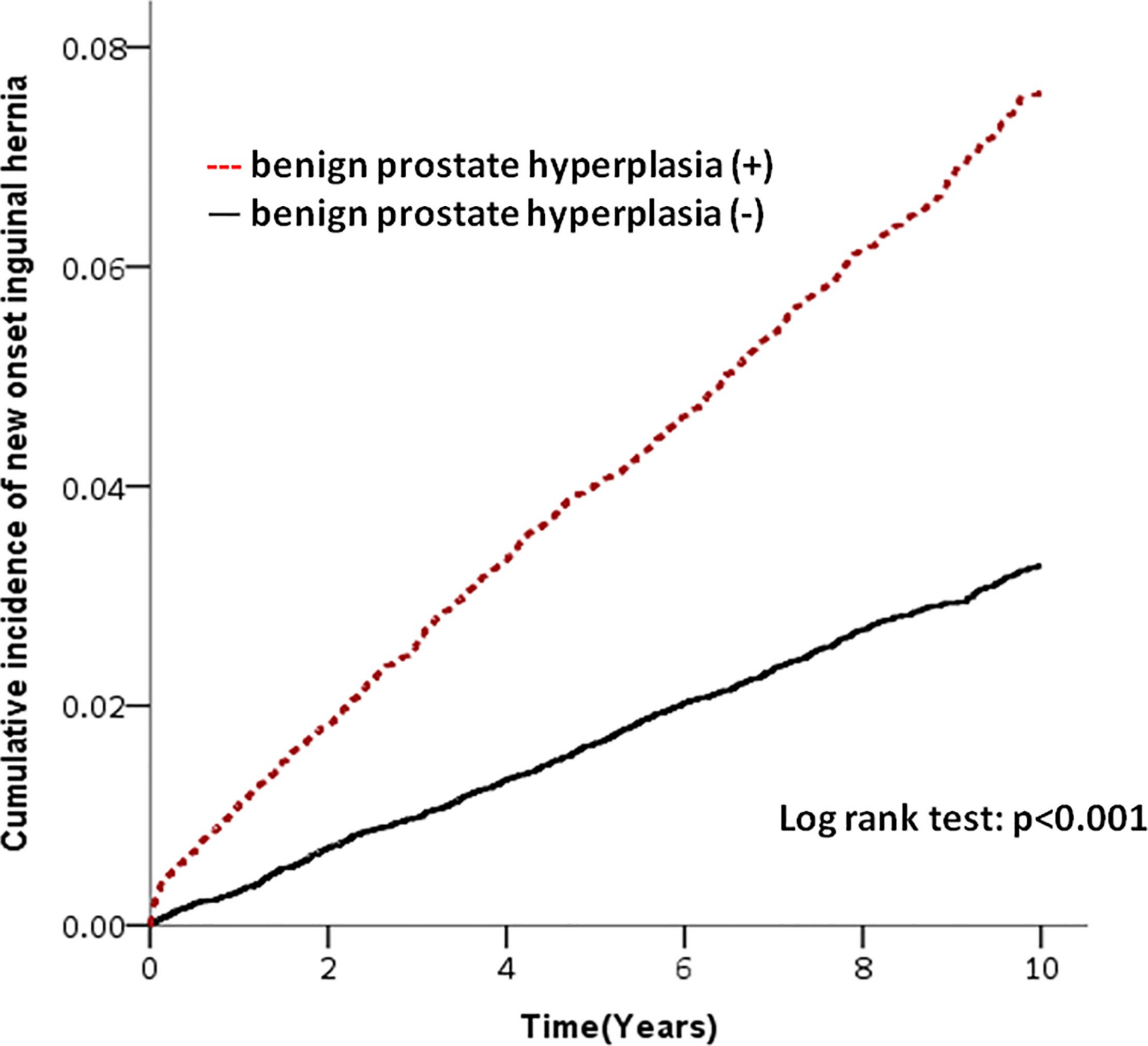
Lower urinary tract symptoms—benign prostatic hyperplasia and the risk of subsequent inguinal hernia. Individuals with lower urinary tract symptoms—benign prostatic hyperplasia were more likely to develop inguinal hernia than those without lower urinary tract symptoms—benign prostatic hyperplasia.

Among the 44,620 sampled individuals, 1,867 (4.18%) developed IH during the 10 years of follow-up, of which 1,303 (5.84%) among men with LUTS-BPH and 735 (2.53%) among men without LUTS-BPH (Fig 1). The incidence rate was 7.65 per 1000 persons and 3.30 per 1000 person years respectively. Subjects with LUTS-BPH were more likely to develop IH than were subjects without LUTS-BPH (crude HR = 2.32; 95% CI = 2.10–2.56, p-value = <0.001) as shown in Table 2. The risk was still increased after adjusting for age and comorbidities (adjusted HR = 2.25, 95% CI = 2.04–2.49, p-value < 0.001). After age stratification, the adjusted HR for subsequent IH was 2.27 (95% CI = 2.05–2.50) in those aged equal to or more than 40 years of age, which showed significant difference (p-value <0.001), and 1.48 (95% CI = 0.59–3.71) in those aged less than 40 years of age, which showed no significant difference (p-value = 0.402). (Table 2)

**Table 2:** The risk of inguinal hernia between LUTS-BPH (-) cohort and LUTS-BPH(+) cohort (N = 44,620).

			Crude model		Adjusted model^a^	
	Case no	Per 1000 person years	hazard ratio (95% CI)	p value	hazard ratio (95% CI)	p value
Overall						
LUTS-BPH(-)	564	3.30	REF.		REF.	
LUTS-BPH(+)	1303	7.65	2.32 (2.10–2.56)	<0.001	2.25 (2.04–2.49)	<0.001
AGE<40, y/o						
LUTS-BPH(-)	8	1.31	REF.		REF.	
LUTS-BPH(+)	11	1.84	1.41 (0.57–3.51)	0.459	1.48 (0.59–3.71)	0.402
AGE> = 40, y/o						
LUTS-BPH(-)	556	3.37	REF.		REF.	
LUTS-BPH(+)	1292	7.87	2.33 (2.11–2.58)	<0.001	2.27 (2.05–2.50)	<0.001

LUTS-BPH, lower urinary tract symptoms—benign prostate hyperplasia; CI, confidence interval; REF., reference.

^a^Adjusted for age and comorbidities.

Table 3 shows the average follow-up duration and average occurrence duration of new onset IH between individuals with and without LUTS-BPH. The average follow-up durations in the LUTS-BPH(-) and LUTS-BPH(+) cohort (mean ± SD) were 7.67 ± 2.81 and 7.63 ± 2.76 years, respectively. There was no significant difference between them. The durations from index date to the date of new onset IH diagnosis in the LUTS-BPH(-) and LUTS-BPH(+) cohorts (mean ± SD) were 4.44 ± 2.75 and 4.02 ± 2.83 years, respectively, which showed significant difference (p-value <0.003).

**Table 3:** Average follow-up duration and average occurrence duration of new onset inguinal hernia between LUTS-BPH (-) and LUTS-BPH(+) cohort (N = 44,620).

	Follow-up duration (years)			New onset IH (years)		
	Mean	(SD)	p value	Mean	(SD)	p value
LUTS-BPH(-)	7.67	(2.81)		4.44	(2.75)	
LUTS-BPH(+)	7.63	(2.76)	0.167	4.02	(2.83)	0.003

LUTS-BPH, lower urinary tract symptoms—benign prostate hyperplasia; SD, standard deviation; IH, inguinal hernia.

## Discussion

Despite many experts attempting to explain the association between LUTS-BPH and hernia formation, the relation between LUTS-BPH and IH might be more complicated and intertwined because both are affected by aging [11, 36]. In this population-based cohort study, we used Taiwanese men to explore the association between LUTS-BPH and the subsequent risk of IH. To our knowledge, this is currently the largest cohort study focusing on the relationship between LUTS-BPH and IH (n = 44,620, including 1,867 men who developed IH patients). We found that preceding LUTS-BPH is an independent risk factor for subsequent IH after controlling for potential confounders (adjusted HR = 2.25, 95% CI = 2.04–2.49, p-value < 0.001). The relationship was prominent in individuals equal to or more than 40 years of age (HR = 2.27, 95% CI = 2.05–2.50, p-value<0.001). Additionally, average duration from diagnosis of LUTS-BPH to discover IH was 4.02 years, which is much shorter than patients without LUTS-BPH.

Our findings are supported with the results of the study published on 2011 by Reis et al, who were the first to investigate the correlation between the presence of IH and LUTS-BPH through quantification by the International Prostate Symptom Score (IPSS) [26]. The IPSS includes seven questions for storage symptoms (frequency, urgency and nocturia) and voiding symptoms (incomplete emptying, intermittency, weak stream and straining) and indicates how LUTS-BPH affects urine void [37]. The study retrospectively categorized 52 patients into one group of LUTS-BPH with IH and one group of LUTS-BPH without IH. The IPSS was significantly higher in those with LUTS-BPH and IH. This provides a good hint about LUTS-BPH being related to IH. Significant increase of PVR in the group of LUTS-BPH and IH also supports the findings [26]. Another study showed that the prevalence of significant LUTS was found to be 48% in patients with IH [38], which also assists our results. Interestingly, a prospective controlled clinical trial analyzed the changes of uroflowmetric parameters following IH repair surgery, which demonstrated that IH repair could significantly affect the maximum flow rate and PVR on postoperative day 1. They concluded that we should identify patients suitable for preoperative treatment before IH repair to reduce the possibility of urinary adverse effects [39].

The mechanisms that might explain the increased risk of subsequent IH in patients with LUTS-BPH are unclear. One possible hypothesis is that LUTS-BPH patients have to push or strain to begin urination, which might lead to an increase in intra-abdominal pressure [37]. Other symptoms, such as frequency, intermittency, incomplete emptying and weak stream may also result in raising intra-abdominal pressure [37]. An extension of these findings recommend that increased intra-abdominal pressure, over time, provides a causal link between benign prostatic hyperplasia, low urinary tract symptoms and inguinal hernia [37].

However, Sentürk achieved contrary conclusions through IPSS in investigating the relation between LUTS-BPH and IH [25]. Per a similar experiment, the patients above 50 years were divided into two groups, LUTS-BPH with IH (n = 50) and LUTS-BPH without IH (n = 50). There was no significant difference in IPSS between the two groups, but prostate volume was significantly enlarged in patients with LUTS-BPH and IH. The result may be accounted for by poor correlation between prostate size and LUTS, and small population size as well. In addition, subclinical inguinal hernia is also an obstacle for us to speculate that LUTS-BPH lead to subsequent IH. A prospective study showed that undiagnosed IH was found accidently in 13% of patients when undergoing laparoscopic surgery [40].

Both LUTS-BPH and IH are not malignant diseases but they indeed impact quality of life. Most men diagnosed with LUTS-BPH suffer from annoying urinary dysfunction and make regular visit to an outpatient clinic for long-term medical control and even surgical intervention [41]. On the contrary, IH may be ignored because it is painless in most circumstances; however, it becomes enlarged and leads to pain over time. Once the intra-abdominal contents become trapped within an inguinal sac, the incarceration or strangulation of intestines might occur. The latter can be fetal and needs surgery immediately [1]. It is noteworthy that our study showed the duration of follow-up since diagnosis of LUTS-BPH to notice the presence of IH is 4.02 years. This reminds clinicians to perform careful groin examinations to discover possible hernia formation in patients visiting for LUTS-BPH medication, thereby allowing progressive inguinal hernia to be treated in a more timely manner.

There are several limitations in our study. Firstly, it is a retrospective analysis. Further studies could consider arranging prospective studies to improve the findings. Secondly, analyzed data was retrieved from the Taiwanese National Health Insurance database, which is a coding system rather than a medical chart review, so clinical information like uroflow data, prostate volume, IPSS, body mass index or detailed treatments could not be determined. Thirdly, although we enrolled as many as possible confounders, some factors were not included in our study, such as smoking status, past history of abdominal surgeries, educational status and income. Fourthly, our population is of majority Han Chinese ethnicity, which may not be the same as other ethnic groups. Fifthly, the censoring in this dataset should be considered because some patients with LUTS-BPH and IH might not have visited a doctor. Sixthly, our study did not use the history of prescriptions to enhance the diagnosis of LUTS-BPH; however, patients with LUTS-BPH might not take medications, and this means we could include those patients with minor LUTS in our study. Finally, we did not restrict the age of patients to be older than 40 years old, which resulted in 699 LUTS-BPH patients younger than 40 years old being enrolled in our study. As we known, lower urinary tract symptoms prevalence in men younger than 40 years old was around 8% [42], so it is worthy of studying whether the chance of getting IH is also increasing in this group of patients. In spite of the limitations mentioned above, this is a large-scale study composed of two groups with similar characteristics and comorbidities. This is also the first nationwide study investigating the relationship between LUTS-BPH and hernia formation in a Taiwanese population. The long-term observational data would assist physicians in keeping IH in mind when treating LUTS-BPH patients.

## Conclusion

Apart from aging, LUTS-BPH is associated with subsequent IH formation. Clinicians should pay attention to groin examination for those men with LUTS-BPH and initiate long-term follow-up.
