# Supplementary material for: Lower urinary tract symptoms–Benign prostatic hyperplasia may increase the risk of subsequent inguinal hernia in a Taiwanese population: A nationwide population-Based cohort study
Source: PLoS One. 2020 Jun 8;15(6):e0234329. doi: 10.1371/journal.pone.0234329 (PMC7279606; doi:10.1371/journal.pone.0234329)
Supplement: Attachment — Submitted filename: Response to Reviewers.docx [file pone.0234329.s001.docx]

Dear editor and reviewers:

We are very grateful to your comments for the manuscript. We also appreciate the time and effort you and each of the reviewers have dedicated to providing insightful feedback on ways to strengthen our paper. Thus, it is with great pleasure that we resubmit our article for further consideration. We have incorporated changes that reflect the detailed suggestions you have graciously provided. We also hope that our edits and the responses we provide below satisfactorily address all the issues and concerns you and the reviewers have noted.

To facilitate your review of our revisions, the following is a point-by-point response to the questions and comments delivered in your letter dated 3/27/2020.

Editor’s Suggestions:

1. In addition to the reviewers comments I ask you to consider changing: 'urine stasis' and 'recurrent infection' (in your introduction) into 'post void residual (PVR)' and 'urinary tract infection'

- RESPONSE: Thank you for this suggestion. We revised these paraphrases showed on line10-11, revised manuscript page 5.

2. I also ask you to better explain how 'the average patient in your country (ICD9 database) is coded BPH (600.XX). E.g. Is a rectal exam always done before this diagnosis (do guidelines direct in this or not? Maybe, related to this, it is possible how a diagnosis of BPH is made for patients <40. Or is there a high chance that patients with voiding symptoms are coded BPH (eg. without 'urinary obstruction'.-see ICD-9 sub-coding).

- RESPONSE: This is a really good point and we will explain it more clearly in the manuscript. In our country, a diagnosis of BPH was based on the doctor’s judgements, either by clinical symptoms and/or digital examinations. There was no unanimous guidelines. So, it’s difficult to know the definite reasons why these patients <40 were diagnosed as BPH. There is a high chance that patients with voiding symptoms are coded BPH, but we did not have further information. We will add this limitation into our discussion and we revised the related paragraph as below: “We used the ninth revision of the International Classification of Diseases (ICD9) codes 600.XX to identify the BPH cohort. A diagnosis of BPH was based on the clinical symptoms and/or digital examinations by physicians.” (line 1 to 4, revised manuscript page 7)

Limitations: “Finally, we did not restrict the age of patients to be older than 40 years old, which resulted in 699 BPH patients younger than 40 years old being enrolled in our study. As we known, lower urinary tract symptoms prevalence in men younger than 40 years old was around 8% [41], and it is worthy of studying whether the chance of getting IH is also increasing in this group of patients.” (line 6 to 10, revised manuscript page 17)

3. Last but not least, I have some difficulty to understand that the 14 days difference in diagnoses can have a pathophysiological meaning; can you slightly more elaborate on the 'sequential' -hypothesis?

- RESPONSE: I apologized that I did not make the **table 3** more clear. The 14 days difference is for follow-up duration, which means the mean follow-up duration for BPH(-) cohort (N=22,310) was 7.67 years and for BPH(+) cohort (N=22,310) was 7.63, and there was no significant difference between them (P=0.167).

The duration from index date to the diagnosis of inguinal hernia was listed in the right side of **Table 3**. The mean duration for BPH(-)IH(+) patients (N=564) was 4.44 years and for BPH(+)IH(+) patients (N=1303) was 4.02 years, which was significant different (P=0.003). This finding could give us another evidence that BPH/LUTS might not only affect the incidence of IH, but also shorten the duration of IH formation.

We revised this paragraph as : “The average follow-up durations in the BPH(-) and BPH(+) cohort (mean ± SD) were 7.67 ± 2.81 and 7.63 ± 2.76 years, respectively. There was no significant difference between them. The durations from index date to the date of new onset IH diagnosis in the BPH(-) and BPH(+) cohorts (mean ± SD) were 4.44 ± 2.75 and 4.02 ± 2.83 years, respectively, which showed significant difference (p-value <0.003).” (line 1 to 7 from the bottom, revised manuscript page 11)

Reviewer 1 Comments:

1. Although the authors included some potential confounders, there are still some more such as education status and income. It is possible that patients with high educational level or high income are likely to seek healthcare due to BPH or IH. They should discuss this limitation.

- RESPONSE: Thank you for this suggestion. We added this into the limitations and revised as follows: “Thirdly, although we enrolled as many as possible confounders, some factors were not included in our study, such as smoking status, past history of abdominal surgeries, educational status and income.” (line 1-4 from the bottom, revised manuscript page 16)

2. This study included male patients over 20 years old. There were 699 patients less than 40 years old with BPH. I think that these patients did not have BPH and the authors should change the inclusion criteria.

- RESPONSE: This is a really good point and we will explain it more clearly in the manuscript. The reason why we enrolled male patients over 20 years old, instead of 40 years old, was due to lower urinary tract symptoms prevalence in men younger than 40 years old was around 8% [41], and it is worthy of studying whether the chance of getting IH is also increasing in this group of patients. Besides, it is hard to change the inclusion criteria at this stage, because the study cohort and the matched cohort need to be re-selected (ratio: 1:1), and all tables and figures need to be changed. In the manuscript, we try to minimize the flaw and divided patients by age <40 and age >= 40, which could show the conditions of each age-group. We will add this into our limitations.

Limitations: “Finally, we did not restrict the age of patients to be older than 40 years old, which resulted in 699 BPH patients younger than 40 years old being enrolled in our study. As we known, lower urinary tract symptoms prevalence in men younger than 40 years old was around 8% [41], and it is worthy of studying whether the chance of getting IH is also increasing in this group of patients.” (line 6 to 10, revised manuscript page 17)

Reviewer 2 Comments:

1. I advise to send this article to statistical/epidemiological review (confounding factors, sample size, methods, etc.)

- RESPONSE: Thank you for this suggestion. I sent this article to epidemiological review and we revised the manuscript by their suggestions. Here we listed some key points they suggested:
- Confounding factors: Thirdly, although we enrolled as many as possible confounders, some factors were not included in our study, such as smoking status, past history of abdominal surgeries, educational status and income.” (line 1-4 from the bottom, revised manuscript page 16)
- Sample size: Normally you select more controls, at least a 3:1 ratio. However, this is not possible to change at this stage.
- Methods: Use the term of cohort study with a matched comparison cohort instead of case-control study. “This is a population-based cohort study, including men with BPH and a matched comparison cohort of men without BPH.” (line 1-2 from the bottom, revised manuscript page 6)
- Provide more information about start and end of follow-up. “All medical claims of men aged 20 and above from 1997-2013 year were collected.” (line 1, revised manuscript page 7)
- All men were followed from the date of diagnosis until death, emigration or end of follow-up (31 December 2013), whichever event came first. (line 3-4 from the bottom, revised manuscript page 7)

2. The objective in introduction is clearly stated, but it is so long.

- RESPONSE: Thank you for this suggestion. We revised the introduction to make it shorter.

3. Profound statistics. Have the authors been counseled by statistical professionals and/or epidemiologists?

- RESPONSE: as comment 1.

4. Are there any confounding factors like body mass index, prior abdominal surgery? Have these confounding factors been excluded?

- RESPONSE: Thank you for this suggestion. We had obesity as one of the confounding factors, instead of body mass index. We did not have the data of body mass index because our database was from coding system rather than a medical chart review. Clinical information like uroflow data, prostate volume and body mass index could not be determined. We will add this into limitation. About the abdominal surgery, the well-documented risk factor of inguinal hernia was radical prostatectomy. So we excluded the patients who had received radical prostatectomy before the index date. We did not have the data of other abdominal surgeries, so we also added into our limitation. “Thirdly, although we enrolled as many as possible confounders, some factors were not included in our study, such as smoking status, past history of abdominal surgeries, educational status and income.” (line 1-3 from the bottom, manuscript page 16)

5. The authors should mention an interesting clinical trial; Unal Y, Kilinc MF. Does inguinal hernia repair affect uroflowmetric parameters? A prospective controlled clinical trial. Low Urin Tract Symptoms. 2019 May;11(3):99-103. doi: 10.1111/luts.12239. Epub 2018 Aug 31.

- RESPONSE: Thank you for this suggestion. We revised the manuscript. “Interestingly, a prospective controlled clinical trial analyzed the changes of uroflowmetric parameters following IH repair surgery, which demonstrated that IH repair could significantly affect the maximum flow rate and PVR on postoperative day 1. They concluded that we should identify patients suitable for preoperative treatment before IH repair to reduce the possibility of urinary adverse effects [38]” (line 9 to 14, manuscript page 14)

CONCLUDING REMARKS: Again, thank you for giving us the opportunity to strengthen our manuscript with your valuable comments and queries. We have worked hard to incorporate your feedback and hope that these revisions persuade you to accept our submission.

Sincerely,

Corresponding Author

JIUN-HUNG GENG

Kaohsiung Municipal Hsiao-Kang Hospital

100 Shih-Chuan 1st Road, Kaohsiung 807, Taiwan

Telephone: +886-7-3208212

E-mail: u9001090@hotmail.com
