# Supplementary material for: Lower urinary tract symptoms–Benign prostatic hyperplasia may increase the risk of subsequent inguinal hernia in a Taiwanese population: A nationwide population-Based cohort study
Source: PLoS One. 2020 Jun 8;15(6):e0234329. doi: 10.1371/journal.pone.0234329 (PMC7279606; doi:10.1371/journal.pone.0234329)
Supplement: Attachment — Submitted filename: Response to Reviewers 2.docx [file pone.0234329.s002.docx]

Dear editor and reviewers:

We are very grateful to your comments for the manuscript.

To facilitate your review of our revisions, the following is a point-by-point response to the questions and comments delivered in your letter dated 4/24/2020.

Editor’s Suggestions:

1. Consider changing the title to: ‘Lower Urinary Tract Symptoms -Benign Prostatic Hyperplasia May Increase the Risk of Subsequent Diagnosis of Inguinal Hernia in a Taiwanese Population: A Nationwide Population-Based Cohort Study’.

- RESPONSE: Thank you for this suggestion. We revised the title of our study. (Page 1, line 1)

2. Where you mention lower urinary tract symptoms, you can add LUTS as the abbreviation and change all BPH for LUTS-BPH where appropriate. You may consider referring to: Lee CL, Kuo HC. Current consensus and controversy on the diagnosis of male lower urinary tract symptoms/benign prostatic hyperplasia. Ci Ji Yi Xue Za Zhi. 2017;29(1):6–11. doi:10.4103/tcmj.tcmj_3_17’ but also to ref 36.

- RESPONSE: Thank you for this suggestion. We revised our manuscript by following the recommended instructions. Please checked the file of “revised manuscript with tract changes.”
- We extracted some corrections here “Benign prostatic hyperplasia (BPH) is one of the most common causes of male lower urinary tract symptoms (LUTS) and occurs with aging [22, Lee CL, Kuo HC]. As LUTS-BPH progresses despite medical treatment, post void residual (PVR) and urinary tract infection can be identified requiring appropriate surgical intervention. (Page 5, line7-10)

3. With my excuse for overlooking this earlier, I ask you also to remove reference 39 and replace that with a reference to 36. 39 is not making this conclusion (and the discussion about the specific medication is irrelevant for your study and hypothetical/speculative (and has in many subsequent studies demonstrated not to have any effect on outflow obstruction)

- RESPONSE: Thank you for this suggestion. We removed reference 39 and replace that with a reference to 36.

CONCLUDING REMARKS: Again, thank you for giving us the opportunity to strengthen our manuscript with your valuable comments and queries. We have worked hard to incorporate your feedback and hope that these revisions persuade you to accept our submission.

Sincerely,

Corresponding Author

JIUN-HUNG GENG

Kaohsiung Municipal Hsiao-Kang Hospital

100 Shih-Chuan 1st Road, Kaohsiung 807, Taiwan

Telephone: +886-7-3208212

E-mail: u9001090@hotmail.com
