# Supplementary material for: Lower urinary tract symptoms–Benign prostatic hyperplasia may increase the risk of subsequent inguinal hernia in a Taiwanese population: A nationwide population-Based cohort study
Source: PLoS One. 2020 Jun 8;15(6):e0234329. doi: 10.1371/journal.pone.0234329 (PMC7279606; doi:10.1371/journal.pone.0234329)
Supplement: Peer review history

# Peer review: Decision Letter 0

**DOI:** 10.1371/journal.pone.0234329.r001

**Authors:** Peter F.W.M. Rosier

27 Mar 2020

PONE-D-20-01358

Benign Prostatic Hyperplasia May Increase the Risk of Subsequent Inguinal Hernia in a Taiwanese Population: A Nationwide Population-Based Cohort Study

PLOS ONE

Dear Dr. Geng,

Thank you for submitting your manuscript to PLOS ONE. After careful consideration, we feel that it has merit but does not fully meet PLOS ONE’s publication criteria as it currently stands. Therefore, we invite you to submit a revised version of the manuscript that addresses the points raised during the review process.

==============================

ACADEMIC EDITOR:

In addition to the reviewers comments I ask you to consider changing: 'urine stasis' and 'recurrent infection' (in your introduction) into 'post void residual (PVR)'  and 'urinarry tract infection'. I also ask you to better explain how 'the average patient in your country (ICD9 database) is coded BPH (600.XX). E.g. Is a rectal exam always done before this diagnosis (do guidelines direct in this or not? Maybe, related to this, it is possible how a diagnosis of BPH is made for patients <40. Or is there a high chance that patients with voiding symptoms are coded BPH (eg. without 'urinary obstruction'.-see ICD-9 sub-coding). Last but not least, I have some difficulty to understand that the 14 days difference in diagnoses can have a pathophysiological meaning; can you slightly more elaborate on the 'sequential' -hypothesis?

We would appreciate receiving your revised manuscript by May 11 2020 11:59PM. When you are ready to submit your revision, log on to https://www.editorialmanager.com/pone/ and select the 'Submissions Needing Revision' folder to locate your manuscript file.

If you would like to make changes to your financial disclosure, please include your updated statement in your cover letter.

To enhance the reproducibility of your results, we recommend that if applicable you deposit your laboratory protocols in protocols.io, where a protocol can be assigned its own identifier (DOI) such that it can be cited independently in the future. For instructions see: http://journals.plos.org/plosone/s/submission-guidelines#loc-laboratory-protocols

Please include the following items when submitting your revised manuscript:

- A rebuttal letter that responds to each point raised by the academic editor and reviewer(s). This letter should be uploaded as separate file and labeled 'Response to Reviewers'.
- A marked-up copy of your manuscript that highlights changes made to the original version. This file should be uploaded as separate file and labeled 'Revised Manuscript with Track Changes'.
- An unmarked version of your revised paper without tracked changes. This file should be uploaded as separate file and labeled 'Manuscript'.

Please note while forming your response, if your article is accepted, you may have the opportunity to make the peer review history publicly available. The record will include editor decision letters (with reviews) and your responses to reviewer comments. If eligible, we will contact you to opt in or out.

We look forward to receiving your revised manuscript.

Kind regards,

Peter F.W.M. Rosier, M.D. PhD

Academic Editor

PLOS ONE

Journal Requirements:

When submitting your revision, we need you to address these additional requirements:

1. Please ensure that your manuscript meets PLOS ONE's style requirements, including those for file naming. The PLOS ONE style templates can be found at http://www.plosone.org/attachments/PLOSOne_formatting_sample_main_body.pdf and http://www.plosone.org/attachments/PLOSOne_formatting_sample_title_authors_affiliations.pdf

2. Thank you for stating the following in the Acknowledgments Section of your manuscript:

"The authors would like to acknowledge the Department of Internal Medicine and the Statistical Analysis Laboratory in the Department of Medical Research at Kaohsiung Medical University Hospital."

We note that you have provided funding information that is not currently declared in your Funding Statement. However, funding information should not appear in the Acknowledgments section or other areas of your manuscript. We will only publish funding information present in the Funding Statement section of the online submission form.

Please remove any funding-related text from the manuscript and let us know how you would like to update your Funding Statement. Currently, your Funding Statement reads as follows:

"The author(s) received no specific funding for this work."

[Note: HTML markup is below. Please do not edit.]

Reviewers' comments:

Reviewer's Responses to Questions

**Comments to the Author**

1. Is the manuscript technically sound, and do the data support the conclusions?

The manuscript must describe a technically sound piece of scientific research with data that supports the conclusions. Experiments must have been conducted rigorously, with appropriate controls, replication, and sample sizes. The conclusions must be drawn appropriately based on the data presented. 

Reviewer #1: Yes

Reviewer #2: Yes

**********

2. Has the statistical analysis been performed appropriately and rigorously? 

Reviewer #1: Yes

Reviewer #2: I Don't Know

**********

3. Have the authors made all data underlying the findings in their manuscript fully available?

The PLOS Data policy requires authors to make all data underlying the findings described in their manuscript fully available without restriction, with rare exception (please refer to the Data Availability Statement in the manuscript PDF file). The data should be provided as part of the manuscript or its supporting information, or deposited to a public repository. For example, in addition to summary statistics, the data points behind means, medians and variance measures should be available. If there are restrictions on publicly sharing data—e.g. participant privacy or use of data from a third party—those must be specified.

Reviewer #1: Yes

Reviewer #2: Yes

**********

4. Is the manuscript presented in an intelligible fashion and written in standard English?

PLOS ONE does not copyedit accepted manuscripts, so the language in submitted articles must be clear, correct, and unambiguous. Any typographical or grammatical errors should be corrected at revision, so please note any specific errors here.

Reviewer #1: Yes

Reviewer #2: Yes

**********

5. Review Comments to the Author

Please use the space provided to explain your answers to the questions above. You may also include additional comments for the author, including concerns about dual publication, research ethics, or publication ethics. (Please upload your review as an attachment if it exceeds 20,000 characters)

Reviewer #1: In this study, the authors investigated the relationship between BPH and subsequent inguinal hernia (IH) in a Taiwanese Population based on the nationwide database. After adjusting for age and related comorbidities, they showed BPH was significantly associated with subsequent IH formation. Their results did not report so new findings. However, this is a large-scale study (n=44620) composed of groups adjusted by the potential confounders and should be considered to be published. There are some issues to be addressed.

1: Although the authors included some potential confounders, there are still some more such as education status and income. It is possible that patients with high educational level or high income are likely to seek healthcare due to BPH or IH. They should discuss this limitation.

2: This study included male patients over 20 years old. There were 699 patients less than 40 years old with BPH. I think that these patients did not have BPH and the authors should change the inclusion criteria.

Reviewer #2: This is a good case control epidemiologic study about the association between Benign Prostate Hiperplasia and Inguinal Hernia.

However, I advise to send this article to statistical/epidemiological review (confounding factors, sample size, methods, etc.)

Title

1. nice and clear

Introduction:

1. The objective is clearly stated, but it is so long.

Materials and Methods

1. Robust study design

2. Profound statistics. Have the authors been counseled by statistical professionals and/or epidemiologists?

3. Are there any confounding factors like body mass index, prior abdominal surgery? Have these confounding factors been excluded?

Results

1. Presented nicely.

2. Clear figure and tables.

Discussion

The authors should mention an interesting clinical trial; Unal Y, Kilinc MF. Does inguinal hernia repair affect uroflowmetric parameters? A prospective controlled clinical trial. Low Urin Tract Symptoms. 2019 May;11(3):99-103. doi: 10.1111/luts.12239. Epub 2018 Aug 31.

**********

6. PLOS authors have the option to publish the peer review history of their article (what does this mean?). If published, this will include your full peer review and any attached files.

If you choose “no”, your identity will remain anonymous but your review may still be made public.

**Do you want your identity to be public for this peer review?** For information about this choice, including consent withdrawal, please see our Privacy Policy.

Reviewer #1: No

Reviewer #2: No

[NOTE: If reviewer comments were submitted as an attachment file, they will be attached to this email and accessible via the submission site. Please log into your account, locate the manuscript record, and check for the action link "View Attachments". If this link does not appear, there are no attachment files to be viewed.]

While revising your submission, please upload your figure files to the Preflight Analysis and Conversion Engine (PACE) digital diagnostic tool, https://pacev2.apexcovantage.com/. PACE helps ensure that figures meet PLOS requirements. To use PACE, you must first register as a user. Registration is free. Then, login and navigate to the UPLOAD tab, where you will find detailed instructions on how to use the tool. If you encounter any issues or have any questions when using PACE, please email us at figures@plos.org. Please note that Supporting Information files do not need this step.

# Author comment: Author response to Decision Letter 0

**DOI:** 10.1371/journal.pone.0234329.r002

17 Apr 2020

Dear editor and reviewers:

We are very grateful to your comments for the manuscript. We also appreciate the time and effort you and each of the reviewers have dedicated to providing insightful feedback on ways to strengthen our paper. Thus, it is with great pleasure that we resubmit our article for further consideration. We have incorporated changes that reflect the detailed suggestions you have graciously provided. We also hope that our edits and the responses we provide below satisfactorily address all the issues and concerns you and the reviewers have noted.

To facilitate your review of our revisions, the following is a point-by-point response to the questions and comments delivered in your letter dated 3/27/2020.

Editor’s Suggestions:

1. In addition to the reviewers comments I ask you to consider changing: 'urine stasis' and 'recurrent infection' (in your introduction) into 'post void residual (PVR)' and 'urinary tract infection'

RESPONSE: Thank you for this suggestion. We revised these paraphrases showed on line10-11, revised manuscript page 5.

2. I also ask you to better explain how 'the average patient in your country (ICD9 database) is coded BPH (600.XX). E.g. Is a rectal exam always done before this diagnosis (do guidelines direct in this or not? Maybe, related to this, it is possible how a diagnosis of BPH is made for patients <40. Or is there a high chance that patients with voiding symptoms are coded BPH (eg. without 'urinary obstruction'.-see ICD-9 sub-coding).

RESPONSE: This is a really good point and we will explain it more clearly in the manuscript. In our country, a diagnosis of BPH was based on the doctor’s judgements, either by clinical symptoms and/or digital examinations. There was no unanimous guidelines. So, it’s difficult to know the definite reasons why these patients <40 were diagnosed as BPH. There is a high chance that patients with voiding symptoms are coded BPH, but we did not have further information. We will add this limitation into our discussion and we revised the related paragraph as below: “We used the ninth revision of the International Classification of Diseases (ICD9) codes 600.XX to identify the BPH cohort. A diagnosis of BPH was based on the clinical symptoms and/or digital examinations by physicians.” (line 1 to 4, revised manuscript page 7) 

Limitations: “Finally, we did not restrict the age of patients to be older than 40 years old, which resulted in 699 BPH patients younger than 40 years old being enrolled in our study. As we known, lower urinary tract symptoms prevalence in men younger than 40 years old was around 8% [41], and it is worthy of studying whether the chance of getting IH is also increasing in this group of patients.” (line 6 to 10, revised manuscript page 17)

3. Last but not least, I have some difficulty to understand that the 14 days difference in diagnoses can have a pathophysiological meaning; can you slightly more elaborate on the 'sequential' -hypothesis?

RESPONSE: I apologized that I did not make the table 3 more clear. The 14 days difference is for follow-up duration, which means the mean follow-up duration for BPH(-) cohort (N=22,310) was 7.67 years and for BPH(+) cohort (N=22,310) was 7.63, and there was no significant difference between them (P=0.167). 

The duration from index date to the diagnosis of inguinal hernia was listed in the right side of Table 3. The mean duration for BPH(-)IH(+) patients (N=564) was 4.44 years and for BPH(+)IH(+) patients (N=1303) was 4.02 years, which was significant different (P=0.003). This finding could give us another evidence that BPH/LUTS might not only affect the incidence of IH, but also shorten the duration of IH formation.

We revised this paragraph as : “The average follow-up durations in the BPH(-) and BPH(+) cohort (mean ± SD) were 7.67 ± 2.81 and 7.63 ± 2.76 years, respectively. There was no significant difference between them. The durations from index date to the date of new onset IH diagnosis in the BPH(-) and BPH(+) cohorts (mean ± SD) were 4.44 ± 2.75 and 4.02 ± 2.83 years, respectively, which showed significant difference (p-value <0.003).” (line 1 to 7 from the bottom, revised manuscript page 11)

Reviewer 1 Comments:

1. Although the authors included some potential confounders, there are still some more such as education status and income. It is possible that patients with high educational level or high income are likely to seek healthcare due to BPH or IH. They should discuss this limitation.

RESPONSE: Thank you for this suggestion. We added this into the limitations and revised as follows: “Thirdly, although we enrolled as many as possible confounders, some factors were not included in our study, such as smoking status, past history of abdominal surgeries, educational status and income.” (line 1-4 from the bottom, revised manuscript page 16)

2. This study included male patients over 20 years old. There were 699 patients less than 40 years old with BPH. I think that these patients did not have BPH and the authors should change the inclusion criteria.

RESPONSE: This is a really good point and we will explain it more clearly in the manuscript. The reason why we enrolled male patients over 20 years old, instead of 40 years old, was due to lower urinary tract symptoms prevalence in men younger than 40 years old was around 8% [41], and it is worthy of studying whether the chance of getting IH is also increasing in this group of patients. Besides, it is hard to change the inclusion criteria at this stage, because the study cohort and the matched cohort need to be re-selected (ratio: 1:1), and all tables and figures need to be changed. In the manuscript, we try to minimize the flaw and divided patients by age <40 and age >= 40, which could show the conditions of each age-group. We will add this into our limitations. 

Limitations: “Finally, we did not restrict the age of patients to be older than 40 years old, which resulted in 699 BPH patients younger than 40 years old being enrolled in our study. As we known, lower urinary tract symptoms prevalence in men younger than 40 years old was around 8% [41], and it is worthy of studying whether the chance of getting IH is also increasing in this group of patients.” (line 6 to 10, revised manuscript page 17)

Reviewer 2 Comments:

1. I advise to send this article to statistical/epidemiological review (confounding factors, sample size, methods, etc.)

RESPONSE: Thank you for this suggestion. I sent this article to epidemiological review and we revised the manuscript by their suggestions. Here we listed some key points they suggested:

- Confounding factors: Thirdly, although we enrolled as many as possible confounders, some factors were not included in our study, such as smoking status, past history of abdominal surgeries, educational status and income.” (line 1-4 from the bottom, revised manuscript page 16)

- Sample size: Normally you select more controls, at least a 3:1 ratio. However, this is not possible to change at this stage.

- Methods: Use the term of cohort study with a matched comparison cohort instead of case-control study. “This is a population-based cohort study, including men with BPH and a matched comparison cohort of men without BPH.” (line 1-2 from the bottom, revised manuscript page 6)

- Provide more information about start and end of follow-up. “All medical claims of men aged 20 and above from 1997-2013 year were collected.” (line 1, revised manuscript page 7)

- All men were followed from the date of diagnosis until death, emigration or end of follow-up (31 December 2013), whichever event came first. (line 3-4 from the bottom, revised manuscript page 7)

2. The objective in introduction is clearly stated, but it is so long.

RESPONSE: Thank you for this suggestion. We revised the introduction to make it shorter.

3. Profound statistics. Have the authors been counseled by statistical professionals and/or epidemiologists?

RESPONSE: as comment 1.

4. Are there any confounding factors like body mass index, prior abdominal surgery? Have these confounding factors been excluded?

RESPONSE: Thank you for this suggestion. We had obesity as one of the confounding factors, instead of body mass index. We did not have the data of body mass index because our database was from coding system rather than a medical chart review. Clinical information like uroflow data, prostate volume and body mass index could not be determined. We will add this into limitation. About the abdominal surgery, the well-documented risk factor of inguinal hernia was radical prostatectomy. So we excluded the patients who had received radical prostatectomy before the index date. We did not have the data of other abdominal surgeries, so we also added into our limitation. “Thirdly, although we enrolled as many as possible confounders, some factors were not included in our study, such as smoking status, past history of abdominal surgeries, educational status and income.” (line 1-3 from the bottom, manuscript page 16)

5. The authors should mention an interesting clinical trial; Unal Y, Kilinc MF. Does inguinal hernia repair affect uroflowmetric parameters? A prospective controlled clinical trial. Low Urin Tract Symptoms. 2019 May;11(3):99-103. doi: 10.1111/luts.12239. Epub 2018 Aug 31.

RESPONSE: Thank you for this suggestion. We revised the manuscript. “Interestingly, a prospective controlled clinical trial analyzed the changes of uroflowmetric parameters following IH repair surgery, which demonstrated that IH repair could significantly affect the maximum flow rate and PVR on postoperative day 1. They concluded that we should identify patients suitable for preoperative treatment before IH repair to reduce the possibility of urinary adverse effects [38]” (line 9 to 14, manuscript page 14)

CONCLUDING REMARKS: Again, thank you for giving us the opportunity to strengthen our manuscript with your valuable comments and queries. We have worked hard to incorporate your feedback and hope that these revisions persuade you to accept our submission.

Sincerely,

Corresponding Author

JIUN-HUNG GENG

Attachment Submitted filename: Response to Reviewers.docx Click here for additional data file.

# Peer review: Decision Letter 1

**DOI:** 10.1371/journal.pone.0234329.r003

**Authors:** Peter F.W.M. Rosier

23 Apr 2020

PONE-D-20-01358R1

Benign Prostatic Hyperplasia May Increase the Risk of Subsequent Inguinal Hernia in a Taiwanese Population: A Nationwide Population-Based Cohort Study

PLOS ONE

Dear Dr. Geng,

Thank you for submitting your manuscript to PLOS ONE. After careful consideration, we feel that it has merit but does not fully meet PLOS ONE’s publication criteria as it currently stands. Therefore, we invite you to submit a revised version of the manuscript that addresses the points raised during the review process.

==============================

ACADEMIC EDITOR: 

Now you have explained that: ‘A diagnosis of BPH was based on the clinical symptoms and/or digital examinations by physicians’, I ask you to consider changing the title to: ‘Lower Urinary Tract Symptoms -Benign Prostatic Hyperplasia May Increase the Risk of Subsequent Diagnosis of Inguinal Hernia in a Taiwanese Population: A Nationwide Population-Based Cohort Study’. Where you mention lower urinary tract symptoms, you can add LUTS as the abbreviation and change all BPH for LUTS-BPH where appropriate. You may consider referring to: Lee CL, Kuo HC. Current consensus and controversy on the diagnosis of male lower urinary tract symptoms/benign prostatic hyperplasia. Ci Ji Yi Xue Za Zhi. 2017;29(1):6–11. doi:10.4103/tcmj.tcmj_3_17’ but also to ref 36. With my excuse for overlooking this earlier, I ask you also to remove reference 39 and replace that with a reference to 36. 39 is not making this conclusion (and the discussion about the specific medication is irrelevant for your study and hypothetical/speculative (and has in many subsequent studies demonstrated not to have any effect on outflow obstruction)

==============================

We would appreciate receiving your revised manuscript by Jun 07 2020 11:59PM. When you are ready to submit your revision, log on to https://www.editorialmanager.com/pone/ and select the 'Submissions Needing Revision' folder to locate your manuscript file.

If you would like to make changes to your financial disclosure, please include your updated statement in your cover letter.

To enhance the reproducibility of your results, we recommend that if applicable you deposit your laboratory protocols in protocols.io, where a protocol can be assigned its own identifier (DOI) such that it can be cited independently in the future. For instructions see: http://journals.plos.org/plosone/s/submission-guidelines#loc-laboratory-protocols

Please include the following items when submitting your revised manuscript:

- A rebuttal letter that responds to each point raised by the academic editor and reviewer(s). This letter should be uploaded as separate file and labeled 'Response to Reviewers'.
- A marked-up copy of your manuscript that highlights changes made to the original version. This file should be uploaded as separate file and labeled 'Revised Manuscript with Track Changes'.
- An unmarked version of your revised paper without tracked changes. This file should be uploaded as separate file and labeled 'Manuscript'.

Please note while forming your response, if your article is accepted, you may have the opportunity to make the peer review history publicly available. The record will include editor decision letters (with reviews) and your responses to reviewer comments. If eligible, we will contact you to opt in or out.

We look forward to receiving your revised manuscript.

Kind regards,

Peter F.W.M. Rosier, M.D. PhD

Academic Editor

PLOS ONE

[Note: HTML markup is below. Please do not edit.]

Reviewers' comments:

Reviewer's Responses to Questions

**Comments to the Author**

1. If the authors have adequately addressed your comments raised in a previous round of review and you feel that this manuscript is now acceptable for publication, you may indicate that here to bypass the “Comments to the Author” section, enter your conflict of interest statement in the “Confidential to Editor” section, and submit your "Accept" recommendation.

Reviewer #1: All comments have been addressed

Reviewer #2: All comments have been addressed

**********

2. Is the manuscript technically sound, and do the data support the conclusions?

The manuscript must describe a technically sound piece of scientific research with data that supports the conclusions. Experiments must have been conducted rigorously, with appropriate controls, replication, and sample sizes. The conclusions must be drawn appropriately based on the data presented. 

Reviewer #1: Yes

Reviewer #2: Yes

**********

3. Has the statistical analysis been performed appropriately and rigorously? 

Reviewer #1: Yes

Reviewer #2: Yes

**********

4. Have the authors made all data underlying the findings in their manuscript fully available?

The PLOS Data policy requires authors to make all data underlying the findings described in their manuscript fully available without restriction, with rare exception (please refer to the Data Availability Statement in the manuscript PDF file). The data should be provided as part of the manuscript or its supporting information, or deposited to a public repository. For example, in addition to summary statistics, the data points behind means, medians and variance measures should be available. If there are restrictions on publicly sharing data—e.g. participant privacy or use of data from a third party—those must be specified.

Reviewer #1: Yes

Reviewer #2: Yes

**********

5. Is the manuscript presented in an intelligible fashion and written in standard English?

PLOS ONE does not copyedit accepted manuscripts, so the language in submitted articles must be clear, correct, and unambiguous. Any typographical or grammatical errors should be corrected at revision, so please note any specific errors here.

Reviewer #1: Yes

Reviewer #2: Yes

**********

6. Review Comments to the Author

Please use the space provided to explain your answers to the questions above. You may also include additional comments for the author, including concerns about dual publication, research ethics, or publication ethics. (Please upload your review as an attachment if it exceeds 20,000 characters)

Reviewer #1: Because the authors answer my questions very well, I think that this article should be published in this journal.

Reviewer #2: Thanks to the authors for their efforts to publish their manuscript.

All my comments have been addressed.

**********

7. PLOS authors have the option to publish the peer review history of their article (what does this mean?). If published, this will include your full peer review and any attached files.

If you choose “no”, your identity will remain anonymous but your review may still be made public.

**Do you want your identity to be public for this peer review?** For information about this choice, including consent withdrawal, please see our Privacy Policy.

Reviewer #1: Yes: Tsuyoshi Majima

Reviewer #2: Yes: MF Kilinc

[NOTE: If reviewer comments were submitted as an attachment file, they will be attached to this email and accessible via the submission site. Please log into your account, locate the manuscript record, and check for the action link "View Attachments". If this link does not appear, there are no attachment files to be viewed.]

While revising your submission, please upload your figure files to the Preflight Analysis and Conversion Engine (PACE) digital diagnostic tool, https://pacev2.apexcovantage.com/. PACE helps ensure that figures meet PLOS requirements. To use PACE, you must first register as a user. Registration is free. Then, login and navigate to the UPLOAD tab, where you will find detailed instructions on how to use the tool. If you encounter any issues or have any questions when using PACE, please email us at figures@plos.org. Please note that Supporting Information files do not need this step.

# Author comment: Author response to Decision Letter 1

**DOI:** 10.1371/journal.pone.0234329.r004

20 May 2020

Dear editor and reviewers:

We are very grateful to your comments for the manuscript. 

To facilitate your review of our revisions, the following is a point-by-point response to the questions and comments delivered in your letter dated 4/24/2020.

Editor’s Suggestions:

1. Consider changing the title to: ‘Lower Urinary Tract Symptoms -Benign Prostatic Hyperplasia May Increase the Risk of Subsequent Diagnosis of Inguinal Hernia in a Taiwanese Population: A Nationwide Population-Based Cohort Study’.

# RESPONSE: Thank you for this suggestion. We revised the title of our study. (Page 1, line 1)

2. Where you mention lower urinary tract symptoms, you can add LUTS as the abbreviation and change all BPH for LUTS-BPH where appropriate. You may consider referring to: Lee CL, Kuo HC. Current consensus and controversy on the diagnosis of male lower urinary tract symptoms/benign prostatic hyperplasia. Ci Ji Yi Xue Za Zhi. 2017;29(1):6–11. doi:10.4103/tcmj.tcmj_3_17’ but also to ref 36.

# RESPONSE: Thank you for this suggestion. We revised our manuscript by following the recommended instructions. Please checked the file of “revised manuscript with tract changes.” We extracted some corrections here “Benign prostatic hyperplasia (BPH) is one of the most common causes of male lower urinary tract symptoms (LUTS) and occurs with aging [22, Lee CL, Kuo HC]. As LUTS-BPH progresses despite medical treatment, post void residual (PVR) and urinary tract infection can be identified requiring appropriate surgical intervention. (Page 5, line7-10)

3. With my excuse for overlooking this earlier, I ask you also to remove reference 39 and replace that with a reference to 36. 39 is not making this conclusion (and the discussion about the specific medication is irrelevant for your study and hypothetical/speculative (and has in many subsequent studies demonstrated not to have any effect on outflow obstruction)

# RESPONSE: Thank you for this suggestion. We removed reference 39 and replace that with a reference to 36.

CONCLUDING REMARKS: Again, thank you for giving us the opportunity to strengthen our manuscript with your valuable comments and queries. We have worked hard to incorporate your feedback and hope that these revisions persuade you to accept our submission.

Sincerely,

Corresponding Author

JIUN-HUNG GENG

Kaohsiung Municipal Hsiao-Kang Hospital

100 Shih-Chuan 1st Road, Kaohsiung 807, Taiwan

Telephone: +886-7-3208212 

E-mail: u9001090@hotmail.com

Attachment Submitted filename: Response to Reviewers 2.docx Click here for additional data file.

# Editor report: Decision Letter 2

**DOI:** 10.1371/journal.pone.0234329.r005

**Authors:** Peter F.W.M. Rosier

26 May 2020

Lower Urinary Tract Symptoms - Benign Prostatic Hyperplasia May Increase the Risk of Subsequent Inguinal Hernia in a Taiwanese Population: A Nationwide Population-Based Cohort Study

PONE-D-20-01358R2

Dear Dr. Geng,

We are pleased to inform you that your manuscript has been judged scientifically suitable for publication and will be formally accepted for publication once it complies with all outstanding technical requirements.

Within one week, you will receive an e-mail containing information on the amendments required prior to publication. When all required modifications have been addressed, you will receive a formal acceptance letter and your manuscript will proceed to our production department and be scheduled for publication.

Shortly after the formal acceptance letter is sent, an invoice for payment will follow. To ensure an efficient production and billing process, please log into Editorial Manager at https://www.editorialmanager.com/pone/, click the "Update My Information" link at the top of the page, and update your user information. If you have any billing related questions, please contact our Author Billing department directly at authorbilling@plos.org.

If your institution or institutions have a press office, please notify them about your upcoming paper to enable them to help maximize its impact. If they will be preparing press materials for this manuscript, you must inform our press team as soon as possible and no later than 48 hours after receiving the formal acceptance. Your manuscript will remain under strict press embargo until 2 pm Eastern Time on the date of publication. For more information, please contact onepress@plos.org.

With kind regards,

Peter F.W.M. Rosier, M.D. PhD

Academic Editor

PLOS ONE

Additional Editor Comments (optional):

Reviewers' comments:

# Editor report: Acceptance letter

**DOI:** 10.1371/journal.pone.0234329.r006

**Authors:** Peter F.W.M. Rosier

29 May 2020

PONE-D-20-01358R2 

Lower Urinary Tract Symptoms - Benign Prostatic Hyperplasia May Increase the Risk of Subsequent Inguinal Hernia in a Taiwanese Population: A Nationwide Population-Based Cohort Study 

Dear Dr. Geng:

I am pleased to inform you that your manuscript has been deemed suitable for publication in PLOS ONE. Congratulations! Your manuscript is now with our production department. 

If your institution or institutions have a press office, please notify them about your upcoming paper at this point, to enable them to help maximize its impact. If they will be preparing press materials for this manuscript, please inform our press team within the next 48 hours. Your manuscript will remain under strict press embargo until 2 pm Eastern Time on the date of publication. For more information please contact onepress@plos.org.

For any other questions or concerns, please email plosone@plos.org. 

Thank you for submitting your work to PLOS ONE.

With kind regards,

PLOS ONE Editorial Office Staff

on behalf of

Dr. Peter F.W.M. Rosier 

Academic Editor

PLOS ONE
